# Resveratrol-Induced AMP-Activated Protein Kinase Activation Is Cell-Type Dependent: Lessons from Basic Research for Clinical Application

**DOI:** 10.3390/nu9070751

**Published:** 2017-07-14

**Authors:** Fan Lan, Karen A. Weikel, Jose M. Cacicedo, Yasuo Ido

**Affiliations:** 1 The First People’s Hospital of Chongqing Liang Jiang New Area, Chongqing 401122, China; lanfan106@gmail.com; 2 Division of Natural Sciences & Mathematics, Boston University College of General Studies, Boston, MA 02215, USA; kaweikel@bu.edu; 3 Department of Medicine, Boston University School of Medicine, Boston, MA 02118, USA; cacicedo@bu.edu

**Keywords:** resveratrol, AMP-activated protein kinase (AMPK), SIRT1, liver kinase B (LKB1)

## Abstract

Despite the promising effects of resveratrol, its efficacy in the clinic remains controversial. We were the first group to report that the SIRT1 activator resveratrol activates AMP-activated protein kinase (AMPK) (Diabetes 2005; 54: A383), and we think that the variability of this cascade may be responsible for the inconsistency of resveratrol’s effects. Our current studies suggest that the effect of SIRT1 activators such as resveratrol may not be solely through activation of SIRT1, but also through an integrated effect of SIRT1-liver kinase B1 (LKB1)-AMPK. In this context, resveratrol activates SIRT1 (1) by directly binding to SIRT1; and (2) by increasing NAD^+^ levels by upregulating the salvage pathway through Nampt activation, an effect mediated by AMPK. The first mechanism promotes deacetylation of a limited number of SIRT1 substrate proteins (e.g., PGC-1). The second mechanism (which may be more important than the first) activates other sirtuins in addition to SIRT1, which affects a broad spectrum of substrates. Despite these findings, detailed mechanisms of how resveratrol activates AMPK have not been reported. Here, we show that (1) resveratrol-induced activation of AMPK requires the presence of functional LKB1; (2) Resveratrol increases LKB1 activity, which involves translocation and phosphorylation at T336 and S428; (3) Activation of LKB1 causes proteasomal degradation of LKB1; (4) At high concentrations (50–100 µM), resveratrol also activates AMPK through increasing AMP levels; and (5) The above-mentioned activation mechanisms vary among cell types, and in some cell types, resveratrol fails to activate AMPK. These results suggest that resveratrol-induced activation of AMPK is not a ubiquitous phenomenon. In addition, AMPK-mediated increases in NAD^+^ in the second mechanism require several ATPs, which may not be available in many pathological conditions. These phenomena may explain why resveratrol is not always consistently beneficial in a clinical setting.

## 1. Introduction

**Figure 1:**
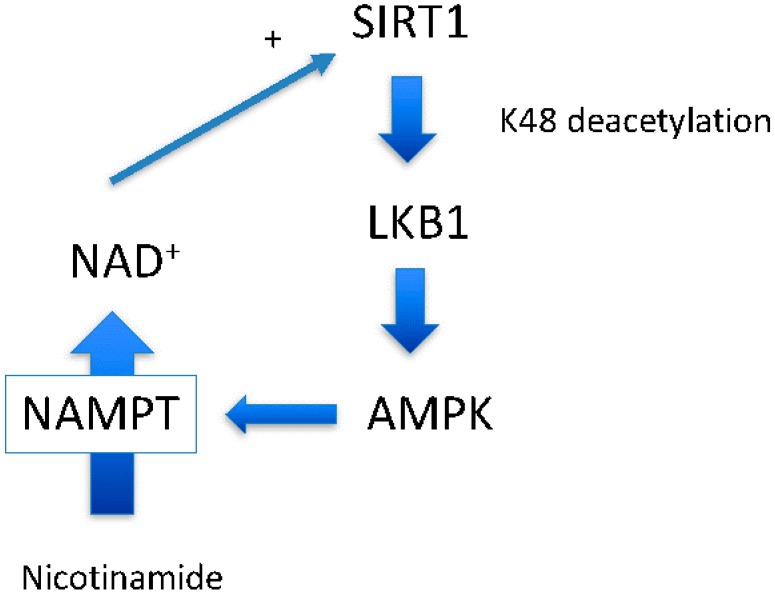
Proposed positive feedback loop to maintain a healthy metabolism. SIRT1-LKB1-AMPK forms a positive feedback loop though NAD^+^ by NAMPT. This loop maintains cell health.

Resveratrol is a well-studied polyphenol that affects cellular physiology in many ways [1]. We became interested in this compound when Sinclair’s group showed that it is an activator of Sir2 and extends life-span in various organisms [2]. We were studying AMP-activated protein kinase (AMPK) at that time [3], and we noticed that SIRT1 (the mammalian ortholog of Sir2) and AMPK are both longevity gene products [4]. Therefore, we hypothesized that SIRT1 and AMPK may have a positive relationship (Figure 1). After testing this hypothesis with resveratrol, we presented our novel results at the American Diabetes Association Scientific Session in 2005 [5]. We then expressed SIRT1 protein ectopically and found that it had the same effect as resveratrol on liver kinase B1 (LKB1) and AMPK activity [6].

However, we noticed that resveratrol-induced activation of AMPK is more complex than that induced by SIRT1 overexpression [7]. This is mainly because, similar to metformin [8], resveratrol inhibits ATP synthase [9,10,11], which can activate AMPK without affecting LKB1 [12]. Regarding the SIRT1 activation, direct activation mechanisms had been questioned by a few groups [13,14,15]. In the end, it seems that resveratrol can bind to SIRT1, but does not change its catalytic activity nor change the Km for NAD^+^ as previously suggested [16]. Instead, Sinclair’s group eventually showed a direct substrate selective activation mechanism in which resveratrol-bound SIRT1 decreases the Km of substrate acetylated lysines in a specific sequence motif [17]. Therefore, only specific acetylated sites of the substrate, such as PGC-1 K778, can be the targets for resveratrol “activation”. Separate groups also reported a similar activation mechanism [18].

**Figure 2:**
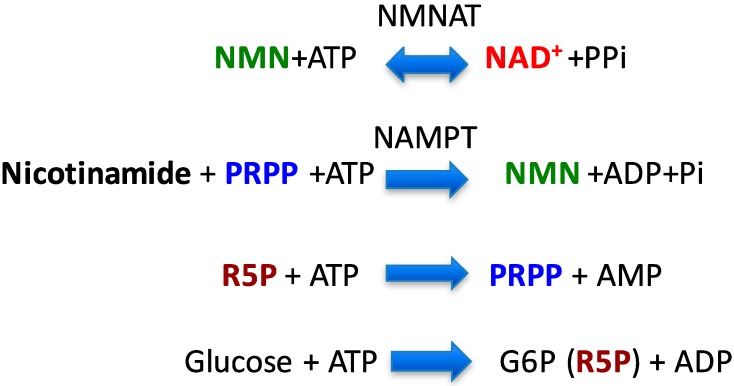
Enzymatic cascade that synthesizes NAD+ from nicotinamide. Nicotinamide can be salvaged to NAD^+^ by consuming several ATPs.

Around the same time at which we demonstrated the SIRT1-LKB1-AMPK activation cascade [6], another group demonstrated that AMPK activation leads to SIRT1 activation by increasing NAD^+^ levels [19], another SIRT1 substrate. Increased NAD^+^ may increase the activity of other sirtuins, including SIRT1 [20], and likely has a greater impact on cellular physiology than direct resveratrol activation of SIRT1 (Figure 1). Indeed, a recent study suggests that there is a close relationship between NAD^+^ levels and longevity through activating various sirtuins [20]. In this scenario, AMPK up-regulates nicotinamide phosphoribosyltransferase (NAMPT) expression [19], the first enzyme of the nicotinamide salvation pathway (Figure 2, the second reaction). With the aid of the second enzyme nicotinamide mononucleotide adenylyltransferase (NMNAT), a nicotinamide is converted to a NAD^+^ [20] (Figure 2, the first reaction). The impact of this reaction is two-fold: it decreases levels of the endogenous SIRT1 inhibitor, nicotinamide, and concomitantly increases the SIRT1 substrate NAD^+^. However, whether this scenario works ubiquitously has not been shown.

In 2005, we found that resveratrol activates AMPK in both a SIRT1- and LKB1-dependent fashion in HEK293 cells [5]. Subsequent experiments showed however that mechanisms of activation of AMPK by resveratrol are strongly cell-type dependent. In this paper, we present detailed mechanisms explaining how resveratrol could activate AMPK and how the activation may vary by cell type. In our hypothesis, resveratrol activation of AMPK is cell-type dependent, and AMPK-induced increases in NAD^+^ levels strongly depends upon the cellular environment, in particular, cytosolic ATP levels. Therefore, when the SIRT1-LKB1-AMPK cascade is active, its effects may vary with pathophysiological circumstances.

## 2. Materials and Methods

### 2.1. Cell Cultures

Human embryonic kidney 293T cells (HEK293T), HeLa CCL-2.1 cells, 3T3 L1 fibroblast and CHO cells were purchased from ATCC (Manassas, VA, USA) and were maintained in OPTI-MEM (Invitrogen; Carlsbad, CA, USA) supplemented with 5% (*v*/*v*) FBS (Hyclone; Logan, UT, USA), and antibiotics. Human umbilical vein endothelial cells (HUVECs) were cultured as previously described [21]. Standard PAGE and western blot was performed as previously described [3]. The following primary antibodies (1:1000 dilution) were used: total AMPK-α, phospho-T172-AMPK and phospho-S428-LKB1 (Cell Signaling Technology; Danvers, MA, USA); ACC (acetyl-CoA Carboxylase), phospho-S79-ACC, (Millipore/Upstate, Charlottesville, VA, USA); β-actin (Sigma, St. Louis, MO, USA); LKB1 (*N*-19, *H*-75, *M*-18, Ley37D/G6) and GST (glutathione *S*-transferase) (Santa Cruz Biotechnologies; Santa Cruz, CA, USA); and phospho-Thr336-LKB1 (ImmuQuest; Ingleby Barwick, Cleveland, UK).

### 2.2. ATP and ADP Assay

Cellular total ATP and ADP were assessed in the cells grown in 6-well pates. After quickly washing with 2 mL ice-cold saline, ATP and ADP were extracted with 500 µL of 1% trichloroacetate (TCA) solution. TCA, which interferes with the assay was removed by washing with an equal volume of ether 5 times, and the remaining solution was lyophilized and stored at −80 °C until the assay. A Luminometric ATP Assay kit (Sigma) was used to measure ATP and ADP, in which ADP was converted to ATP after destroying ATP by ATP-sulfurylase (Sigma) as described previously [22].

### 2.3. cDNAs, Plasmids, Recombinant Viruses

cDNA and plasmids used this study was published previously [6].

Kinase dead LKB1 (D194A) was created by PCR with a primer pair GTGGCACCCTCAAAATCTCCGCCCTGGGCGTGGCCGAGGC (forward) and GCCTCGGCCACGCCCAGGTCGGAGATTTTGAGGGTGCCAC (reverse) and S428A mutant was with a primer pair AAGATCCGCCGGCTGGCGGCCTGCAAGCAGCAG (forward) and CTGCTGCTTGCAGGCCGCCAGCCGGCGGATCTT (reverse) using human pENTR-LKB1 (BC019334) as a template. The mutation was confirmed by sequence analysis. The lentivirus expression vector was created by Gateway LR recombinase (Invtirogen) using a pLent-6 destination vector. LKB1 GST-fusion protein was similarly created using a pDEST-27 vector and transfected to HEK293T cells. Lentiviruses and adenoviruses were prepared as published previously.

### 2.4. Other Procedures

LKB1 activity assay and GFP-LKB1 localization analysis were performed as published previously [6].

### 2.5. Statistical Analysis

Statistical analyses were done by the GLM procedure using SAS (Cary, NC, USA). All results are expressed as means ± standard error (SE). *p* < 0.05 was taken as significant. All experiments were performed 2–4 times to confirm reproducibility.

## 3. Results

### 3.1. Resveratrol-Induced AMPK Activation Requires Functional LKB1 and SIRT1 Activity

**Figure 3:**
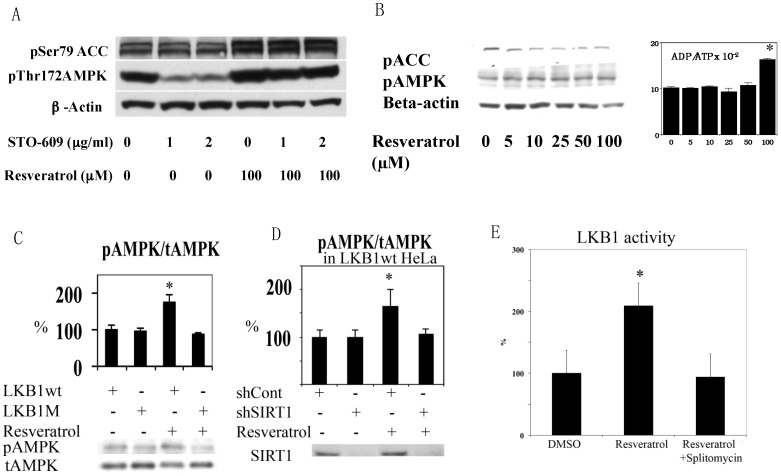
(**A**) Resveratrol-induced activation of AMPK. HEK293 cells were stimulated by 100 µM resveratrol in the presence of the indicated concentrations of CamKK inhibitor STO-609. AMPK activation was clearly seen with inhibition of CamKK (compare lanes 2 vs. 5 and lanes 3 vs. 6); (**B**) Resveratrol failed to activate AMPK in HeLa cell. HeLa cells were stimulated with the indicated concentrations of resveratrol for 1 h. Although 100 µM resveratrol increased the ADP/ATP ratio (*n* = 3), it failed to change AMPK activity; (**C**) HeLa cells with wt LKB1 restore AMPK activation by resveratrol. HeLa cells were infected with lentivirus expressing wild type (wt) or K194A (kinase dead) mutant (M) LKB1 and stimulated with 100 µM resveratrol for 1 h (*n* = 3); (**D**) Resveratrol activation of AMPK is SIRT1 dependent. HeLa cells infected with wt LKB1 were infected with scramble shRNA (shCont) or SIRT1 shRNA (shSIRT1), and were stimulated with 100 µM resveratrol for 1 h (*n* = 3); (**E**) Resveratrol activates LKB1 kinase activity, which was prevented by the Sir2 inhibitor splitomycin. Human umbilical vein endothelial cells were incubated with 100 µM resveratrol or 100 µM resveratrol + 50 µM splitomycin for 1 h. The whole cell lysate was immunoprecipitated with LKB1 antibody and kinase activity was measured with LKBtide and ^32^*P*-ATP. 100 µM resveratrol increased kinase activity by 100% (*n* = 3, * *p* < 0.05) and this was prevented by splitomycin.

We first noticed that resveratrol was not able to increase AMPK T172 phosphorylation clearly in HEK293T cells in normal culture conditions. After using STO609, a calmodulin-dependent kinase kinase (CamKK) inhibitor [23], it was clear that this was because activity of basal CamKK, another upstream kinase of AMPK [24], was too strong and masked the effects of resveratrol (Figure 3A). This result excludes CamKK as a candidate AMPK kinase activated by resveratrol. Although TAK1 (transforming growth factor beta activated protein kinase 1) has been shown to phosphorylate AMPK [25], its activation leads to NFkB activation that does not occur in the presence of resveratrol [26]. Thus, LKB1 is likely the responsible AMPK kinase. Since HeLa cells lack functional LKB1 [27], we tested resveratrol in HeLa cells. Resveratrol increased the ADP/ATP ratio at 100 µM, but still failed to activate AMPK (Figure 3B). We then introduced wild type (wt) and kinase dead (D194A) mutants [6] of LKB1 into HeLa cells by lentivirus infection and tested the effects of resveratrol on AMPK. As shown in Figure 3C, only wt LKB1 is able to activate AMPK. Similar LKB1 dependency was observed in A549 cells that also lack LKB1 (data not shown). Next, we examined SIRT1 dependency in LKB1 wt knocked-in HeLa cells by using lentivirus expressing shRNA to suppress SIRT1 (by more than 80%). Resveratrol failed to activate AMPK in these cells (Figure 3D), suggesting that LKB1-mediated AMPK activation is SIRT1 dependent.

### 3.2. Resveratrol Increases LKB1 Activity, Increases S428, T336 Phosphorylation, and Promotes LKB1 Translocation

**Figure 4:**
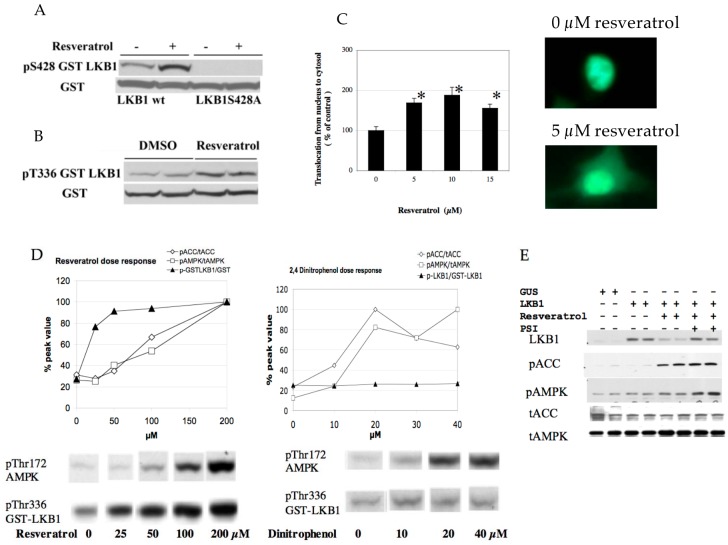
(**A**) Resveratrol increases S428 phosphorylation. Wild type and S428A mutant LKB1-GST plasmid was transfected into HEK293T cells and stimulated by 20 µM resveratrol for 1 h; (**B**) Resveratrol increases T336 phosphorylation. Wild type LKB1-GST plasmid was transfected into HEK293T cells. Cells were treated with either DMSO or 20 µM resveratrol for 1 h; (**C**) Resveratrol induced LKB1 translocation. GFP-LKB1 fusion plasmid was transfected into HEK293T cells and stimulated with the indicated concentrations of resveratrol for 1 h. GFP localization was monitored and calculated as published previously [6]; (**D**) Comparison of resveratrol and dinitrophenol on pT336 LKB1 and pT172 AMPK. GST-LKB1 fusion plasmid was transfected into HEK293T cells and stimulated with the indicated concentrations of resveratrol or dinitrophenol for 1 h. Resveratrol but not dinitrophenol increased pT336 LKB1. pT336 LKB1 was apparent at a lower dose than pT172 AMPK and pS79ACC; (**E**) Resveratrol destabilizes LKB1 which is protected by a proteasome inhibitor. Human umbilical vein endothelial cells were infected with wild type LKB1 (lanes 3–8) and treated with 100 µM resveratrol for 1 h. Resveratrol’s decrease of LKB1 expression (lanes 5–6 vs. 3–4) can be prevented by the addition of 50 µM proteasome inhibitor. Addition of proteasome inhibitor increased pAMPK.

Although resveratrol requires LKB1 activity to phosphorylate AMPK, resveratrol may not alter LKB1 activity. For example, AMPK activation by AICAR or phenformin failed to change LKB1 activity [28]. However, we demonstrated previously that SIRT1 overexpression led to increased LKB1 activity by deacetylation [6]. Similarly, resveratrol increased LKB1 activity as measured in an in vitro kinase assay after immunoprecipitation (Figure 3E). This effect was not observed when the SIRT1 inhibitor splitomycin [29] was added simultaneously (Figure 3E). Splitomycin alone has no direct effects on LKB1 activity in vitro (Supplementary Figure S1). Resveratrol also increased both S428 (Figure 4A) and T336 (Figure 4B) phosphorylation of LKB1. The latter phosphorylation site is autophosphorylated by LKB1 itself and increased when LKB1 forms an active complex with STRAD and MO25 [30]. S428 phosphorylation appears to takes place when LKB1 is translocated to the cytosol (unpublished observation). Corresponding to these data, LKB1 translocation from the nucleus to the cytosol was increased by resveratrol (Figure 4C). Taken together, these results are consistent with a scenario that SIRT1 activation -> LKB1 deacetylation [6] -> LKB1 translocation to the cytosol -> formation of an active LKB1 complex. We then compared resveratrol to 2,4-dinitrophenol (DNP) in activation of AMPK and LKB1 to test whether resveratrol’s effect is unique in activation of LKB1. DNP dissipates the proton gradient in mitochondria so that it decreases cellular energy levels, which activates AMPK but should not activate LKB1. Both resveratrol and DNP are able to activate AMPK, but only resveratrol increased LKB1 T336 phosphorylation (Figure 4D). This indicates that resveratrol can activate LKB1 by an energy independent process.

### 3.3. Resveratrol May Accelerate LKB1 Degradation by the Proteasome

In HUVEC cells, overexpression of LKB1 by lentivirus alone had very little effect on basal AMPK activity (Figure 4E). However, resveratrol-mediated AMPK phosphorylation was more easily seen. Surprisingly, we noted that LKB1 protein expression after resveratrol treatment was strongly diminished (Figure 4E). Although the LKB1 degradation cascade is not known precisely, we suspected that this might be mediated by the proteasome. Indeed, concomitant treatment with the proteasome inhibitor (PSI) prevented LKB1 degradation and AMPK activation became stronger (Figure 4E). This result suggests that resveratrol may reduce LKB1 levels after activation.

### 3.4. Resveratrol-Mediated AMPK Activation Was Not Ubiquitously Observed in Different Cell Types

**Figure 5:**
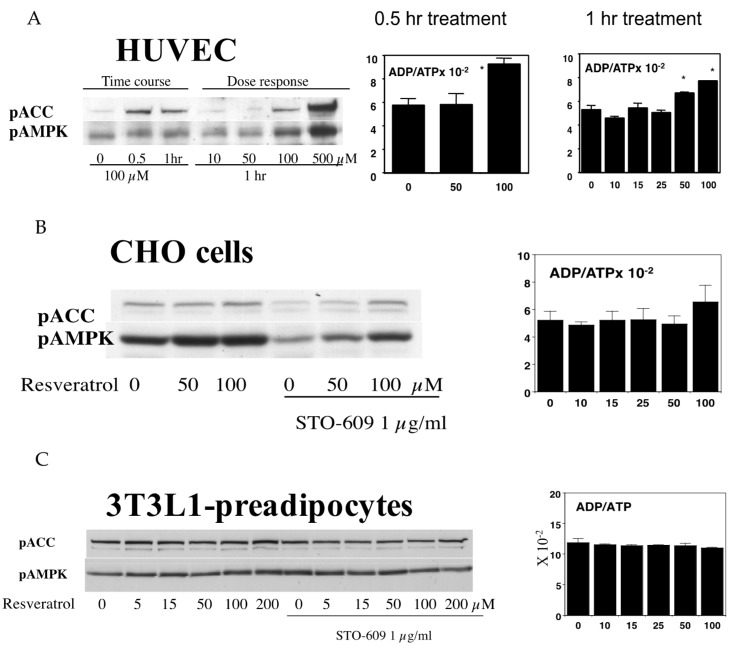
(**A**) Activation of AMPK by resveratrol in HUVEC. HUVECs were incubated in the indicated concentrations of resveratrol for either 0.5 or 1 h. Western blotting analysis shows that the phosphorylation of both ACC and AMPK was increased by 100 µM resveratrol. In the 0.5 h treatment, 100 µM but not 50 µM resveratrol increased the ADP to ATP ratio (*n* = 3, *p* < 0.05). In the 1 h treatments, both doses of resveratrol significantly increased this ratio (*n* = 3, *p* < 0.05), and this was found to be due to increased ADP levels as the ATP levels were unaffected; (**B**) Effects of resveratrol on AMPK in CHO cells. CHO cells were stimulated by the indicated concentrations of resveratrol for 1 h. In some wells, the cells were incubated with 1 µg/mL of the CamKK inhibitor, STO-609, prior to resveratrol stimulation. The inhibition of CamKK decreased the basal ACC and AMPK phosphorylation levels, but the addition of resveratrol increased phosphorylation. Despite increases in the pAMPK and pACC levels, the ADP to ATP ratio was not affected at any of the doses tested (*n* = 3); (**C**) Effects of resveratrol on AMPK in 3T3L-1 preadipocytes. In 3T3-L1 preadipocytes, a resveratrol dose of up to 200 µM was found to be ineffective in increasing the phosphorylation of either AMPK or ACC. Similarly, the ADP/ATP ratio was unaffected by resveratrol in these cells (*n* = 3).

We then asked whether this activation mechanism and its effects are ubiquitous in other cell types. We previously published that, in human primary keratinocytes, as little as 10 µM resveratrol can activate AMPK in 1 h [31]. In human endothelial cells, the activation takes place at the same concentrations that reduce cellular energy levels (50–100 µM), which may take 1 h to achieve (Figure 5A). In Chinese hamster ovary cells (CHO cells), resveratrol activated AMPK without a significant decrease in cellular energy levels (Figure 5B). In 3T3 L1 preadipocytes, resveratrol failed to activate AMPK even at 200 µM, and did not alter energy levels (Figure 5C). On the other hand, in differentiated 3T3L1 adipocytes, resveratrol can activate AMPK at 100 µM even in the presence of a CamKK inhibitor (data not shown). These results suggest that although resveratrol may activate AMPK through both energy-dependent and -independent cascades, the machinery participating in activation may not be similarly operated in every type of cell.

### 3.5. A Potential Method to Increase Resveratrol Activation of AMPK Based on the Above Findings

**Figure 6:**
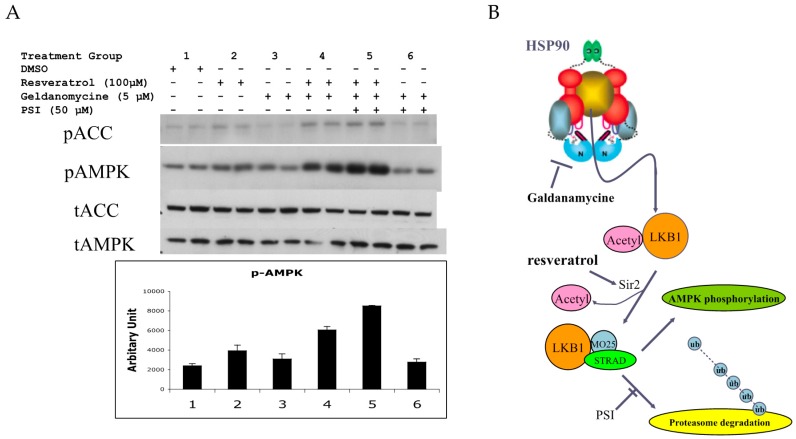
(**A**) Effects of geldanamycin and proteasome inhibitor (PSI) on resveratrol-induced AMPK activation in human umbilical vein endothelial cells (HUVECs). HUVECs were stimulated with 100 µM resveratrol for 1 h with/without 5 µM geldanamycin and/or 50 µM PSI. Geldanamycin with/without PSI alone had no effects on pAMPK (groups 1 vs. 3 and groups 1 vs. 6). Increased pAMPK by resveratrol (groups 1 vs. 2) was intensified with geldanamycin (group 4) and with both geldanamycin and PSI (group 5). * *p* < 0.05 (*n* = 3); (**B**) Current model to activate LKB1 and AMPK by resveratrol. LKB1 is normally acetylated and the majority of LKB1 protein is protected by HSP90. When released from HSP90 bound to STRAD and MO25, LKB1 becomes an active kinase that is able to phosphorylate AMPK. Deacetylated LKB1 may be more susceptible to ubiquitination and degradation. Increased AMP and ADP keeps pAMPK levels high by inhibiting de-phosphorylation of pAMPK. Resveratrol can activate SIRT1 which can deacetylate LKB1, allowing for increased formation of its active complex with STRAD and MO25. In addition, resveratrol may increase AMP and ADP to keeps AMPK active. Inhibition of HSP90 and PSI will enhance resveratrol’s action to activate AMPK.

LKB1 is a client protein of HSP90 [32,33]. This may be related to the fact that LKB1 protein has difficulty folding properly in *E. coli* and even in insect cells. Therefore, chaperone proteins may ensure proper folding. An HSP90 inhibitor such as geldanamycin [33] would release a client protein by inhibiting ATPase activity, and this may increase the amount of LKB1 available for complex formation. We investigated this possibility in normal HUVEC. As shown in Figure 5A, resveratrol slightly increased pAMPK. Geldanamycin alone had little effect, while the combination of resveratrol and geldanamycin increased *p*-AMPK more than resveratrol alone. The addition of proteasome inhibitor (PSI) increased pAMPK further, while PSI itself had a negligible effect (Figure 6. In summary, resveratrol’s effect on AMPK activation could be enhanced by combining HSP90 inhibition and proteasome inhibition. Figure 6B shows a potential cascade in which LKB1 is activated by resveratrol, an HSP90 inhibitor and PSI.

## 4. Discussion

### 4.1. Resveratrol Activates AMPK by Co-Operative Action of Two Mechanisms

Clinically used biguanides such as metformin are known to activate AMPK through LKB1 [28]. Although it was suggested that metformin increases AMP levels [8], many studies failed to show a changing energy status as stated before [34]. This is mainly due to the inability to detect free (bioavailable) AMP by enzymatic, chromatographic or mass spectrometric methods because its levels are too low. The available method to detect free AMP utilizes ^31^*P*-NMR with adenylate kinase and creatine kinase equilibration [34], and this equilibration occurs only in muscle cells. Thus, in the perfused rat heart, metformin clearly increases free AMP levels without changing total ATP, ADP and AMP levels [34]. Since biguanides do not alter LKB1 activity, their activation is solely though energy-dependent mechanisms [28].

In this paper, we showed that resveratrol could activate AMPK via at least two mechanisms: energy-dependently (ATP synthase inhibition) and energy-independently (SIRT1-LKB1 activation). Since both pathways require a functional LKB1, it is not easy to distinguish them. In HUVECs for example, only high concentrations of resveratrol that decrease energy levels increase pAMPK. This appears to indicate that the energy-independent mechanism is not involved. However, resveratrol can also increase LKB1 activity in this cell. Activation of LKB1 as shown in increased phosphorylation of S485 and T336 may occur at as low a concentration as 10–15 µM. However, in the cell types we tested here, this concentration of resveratrol did not cause detectable increases in pAMPK. According to the current activation model of AMPK, albeit small, increased AMP is required for an increase in AMPK phosphorylation through phosphatase resistance mechanisms [35,36]. 10–15 µM resveratrol probably does not lower energy levels at least in short time. We propose that resveratrol-mediated AMPK activation is a co-operation of both mechanisms, which could be an advantage for resveratrol. Thus, in cells such as 3T3L-1 preadipocytes (fibrocytes), resveratrol failed to activate AMPK likely because there was no alteration in energy status even at 200 µM. We know that this cell has SIRT1 and LKB1. So, this cell appears to be resistant to changing energy levels.

It was suggested that resveratrol works as a cAMP specific phosphodiestrase 4 inhibitor to activate SIRT1 and AMPK through CamKK [37]. This is an interesting observation, and this cascade may contribute to some of the effects of resveratrol in AMPK activation. However, the following observations contradict this scenario: (1) resveratrol activated AMPK in the presence of CamKK inhibitor, and (2) resveratrol had no effect on AMPK in HeLa cells (ATCC CCL 2.1) and A549 cells both of which lack LKB1 but have CamKK. In addition, we have not observed any pAMPK increase in differentiating 3T3-L1 cells that were incubated with 0.5 mM isobutylmethylxantine, a general phosphodiesterase inhibitor, to increase cAMP. Our second observation regarding HeLa cells may need special attention, since it directly contradicts the observations of Park and colleagues [37]. This discrepancy may be related to the subtype of HeLa cells available. ATCC provides 3 HeLa cells, CCL2, CCL2.1 and CCL2.2 (S3). We found that sensitivity in CamKK-mediated AMPK activation was different among these cells. CCL2.2 (S3) appears to be the most sensitive to fluctuation of Ca^2+^ levels which even reacts to AICAR that should not activate AMPK (unpublished observation).

### 4.2. LKB1 Deacetylation by SIRT1 Increases Activity but Decreases Stability

In our model, activated AMPK increases cytosolic NAD^+^ levels which leads to activation of SIRT1, and this activates LKB1 leading to activation of AMPK; forming a positive feedback circuit [38] (Figure 1). Since positive feedback cannot be operative to maintaining homeostasis under normal physiology, there should be a point of negative regulation. We think that LKB1 is the point of negative regulation in this system. Previously, during the study of SIRT1 in HepG2 cells, we noted that SIRT1 knockdown increased total LKB1 levels but decreased pLKB1 [39]. Overexpression of SIRT1 showed the opposite effects. In mouse adipose tissues, there was a clear positive correlation between LKB1 acetylation levels and total LKB1 levels but negative correlation between acetylation and pS428 and pT336 LKB1 [39]. This indicates that acetylation of LKB1 stabilizes LKB1 and deacetylation does opposite. The results of the current study agree with these previous observations and further elucidate the mechanism: resveratrol increased LKB1 activity by SIRT1-mediated deacetylation but accelerated proteasome degradation of LKB1. Proteasomal degradation of proteins is typically induced by protein ubiquitination of a lysine residue. This phenomenon is likely initiated by a still-unknown ubiquitin ligase targeting non-acetylated lysine residues of LKB1 [33]. Although some AMPK family protein kinases have been shown to contain ubiquitination motifs, LKB1 does not [40]. LKB1 degradation is likely mediated by K48 ubiquitination but not by K63 ubiquitination as recently reported [41].

### 4.3. Feeding and Starvation Cycles May Regulate the LKB1 Acetylation Cycle

LKB1’s acetylation enzyme has not yet been identified. We observed that p300 can acetylate LKB1 in vitro (unpublished observation). In substrate level regulation, cytosolic/nuclear acetyl-CoA levels may be key in determining LKB1 acetylation status. Cytosolic acetyl-CoA is produced by citrate lyase from citrate that comes from mitochondria when enough substrates (oxaloacetate + acetyl-CoA) and NADH are fueled into it. Thus, normal feeding and starvation cycles may produce a physiological oscillation of LKB1 deacetylation and acetylation, as does AMPK and NAD^+^. Currently, CLOCK gene-mediated circadian oscillation of sirtuins through NAMPT expression and NAD^+^ has been proposed [42]. Our model may operate similarly.

### 4.4. AMPK Activation May Not Always Increase NAD^+^

**Figure 7:**
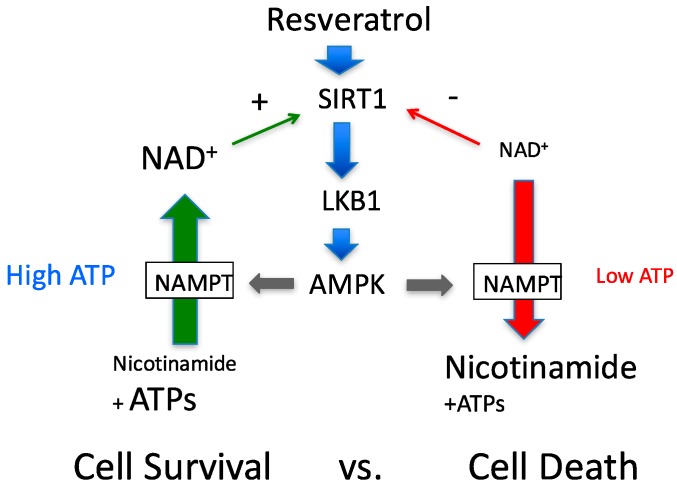
Revised proposed feedback loop to determine cell survival and cell death. Since NAD^+^ synthesis requires several ATPs, ATP availability will determine the effects of the feedback loop. Different amounts ATP, nicotinamide and NAD^+^ are presented using different font sizes.

AMPK activation has been shown to increase expression of NAMPT [19], and, together with NMNAT, increases cellular NAD^+^ levels by salvaging nicotinamide. Although this scenario works under normal physiology, there is caveat in this scenario under pathophysiological conditions. As shown in Scheme II, the whole cascade, including NAMPT, requires a number of ATPs. The NAMPT reaction is weakly coupled to ATP hydrolysis which captures nicotinamide more efficiently [43]. This indicates that the reaction depends on cytosolic ATP availability. Indeed, it was shown in hepatocytes that cytosolic NAD^+^ content strictly depends on cytosolic ATP concentration [44]. Devin and colleagues used 2,4-dinitrophenol, which activates AMPK as we showed in this paper, to titrate ATP levels. The authors clearly showed that lower ATP levels caused lower NAD^+^ levels [44]. Supporting this observation, it was demonstrated that the NMNAT reaction is completely reversible; ATP can be produced at the expense of NAD^+^ [45]. This indicates that although AMPK activation may increase NAMPT expression and activity, this will not automatically increase NAD^+^ levels. Rather, NAD^+^ levels may decrease if there is not sufficient ATP available to initiate the NMNAT reaction (Figure 2). The further decrease in NAD^+^ will reduce cell viability by inhibiting glycolytic flux and ATP production. We think that this phenomenon determines whether AMPK works in cell survival or cell death signaling (Figure 7). In type 2 diabetes, AMPK activation is impaired and cellular ATP is normal. Thus, resveratrol would work to normalize functions by activating SIRT1, AMPK and sirtuins through NAD^+^. For cancer treatment purposes, if the cellular energy production is already compromised, resveratrol may accelerate cell death.

### 4.5. Potential Method to Improve Resveratrol Efficacy

Given the discussion above, the question is when and how resveratrol should be administered. Firstly, as shown in this study, the effect of resveratrol on AMPK could be enhanced by HSP90 inhibition and proteasome inhibition. A literature search showed that epigallocatechin gallate (EGCG), a green tea polyphenol, has both effects [46,47]. So, resveratrol may be more effective with EGCG. Secondly, it may be necessary to increase total LKB1 levels by maintaining acetylation until activation, which likely occurs during the feeding period. Thus, resveratrol may be given during starvation. Thirdly, if the purpose of resveratrol is to increase survival of a cell whose energy levels are already compromised, an initial step of treatment may not be resveratrol but, instead, giving nicotinamide riboside [48]. Nicotinamide riboside can be converted to NAD^+^ by nicotinamide riboside kinase 2 and adenyltransferase using only one ATP [48]. Thus, it may restore the NAD^+^ level without consuming many ATPs. Increased cytosolic NAD^+^ levels will increase glycolytic flux and further restore cellular ATP levels. After restoration of ATP levels, resveratrol will be effective. Finally, we could also restore NAD^+^ levels by increasing PRPP (phophoribosyl pyrophosphate), a substrate of NAMPT (Figure 2). PRPP is derived from the pentose phosphate pathway. The flux from glycolysis to this pathway can be increased by a thiamine mimetic, Benfotiamine. Benfotiamine was introduced by Brownlee et al. [49] to prevent diabetic complications. Their group proposed that excessive glycolytic intermediates which are mediators of glycotoxicity can be diverted to the pentose phosphate cascade by activating transketolase with Benfotiamine. Thus, this may help to increase NAD^+^ synthesis, although alone it did not work in preventing diabetic complications in humans [50]. On the other hand, activation of this cascade may not be good for cancer treatment, as this enzyme is already overexpressed in many cancers [51].

## 5. Conclusions

In summary, we showed the detailed mechanism by which resveratrol activates AMPK through activation of the SIRT1-LKB1-AMPK feedback loop, and discussed how this feedback loop may work. When cytosolic ATP is at normal levels, this activation will induce a variety of signals via LKB1 and sirtuins that can cope with stress. If ATP levels are already diminished, the effect could be the opposite. According to our model, cellular conditions need to be considered during resveratrol administration.

## Supplementary Materials

The following are available online at www.mdpi.com/2072-6643/9/7/751/s1, Figure S1: Effects of various reagents on LKB1 activity in vitro.

## Abbreviations

AMPK	AMP (adenosine monophosphate)-activated protein kinase
EGCG	epigallocatechin gallate
LKB1	liver kinase B1
NAMPT	nicotinamide phosphoribosyltransferase
NMN	nicotinamide mononucleotide
NMNAT	nicotinamide mononucleotide adenylyltransferase
PPi	pyrophosphate; PRPP: phophoribosyl pyrophosphate
PSI	proteasome inhibitor
