# Supplementary figures and images for: Resveratrol-Induced AMP-Activated Protein Kinase Activation Is Cell-Type Dependent: Lessons from Basic Research for Clinical Application
Source: Nutrients. 2017 Jul 14;9(7):751. doi: 10.3390/nu9070751 (PMC5537865; doi:10.3390/nu9070751)

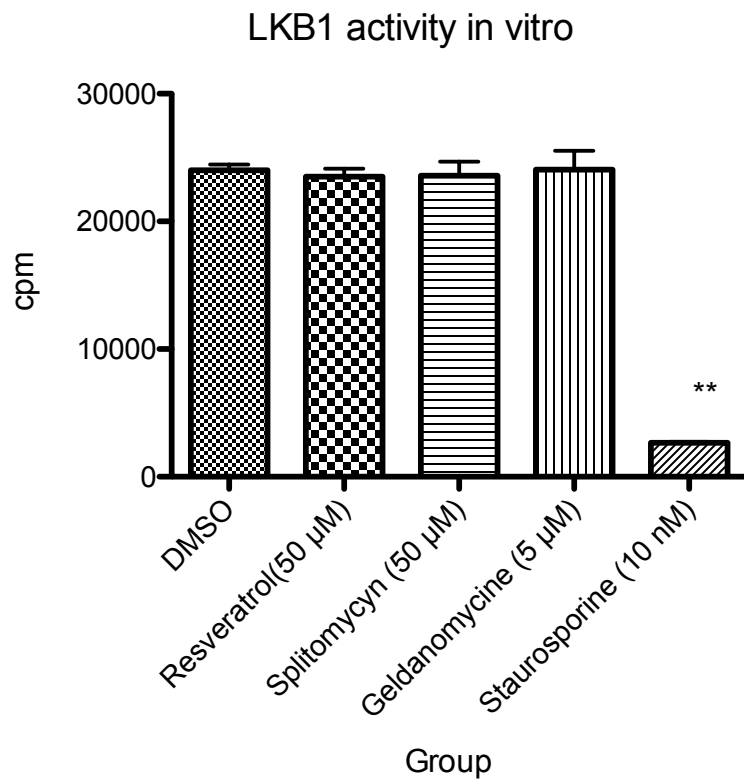

**pT336-LKB1**

Flag-MO25

His- STRAD

**GST-LKB1**

**Transfection**

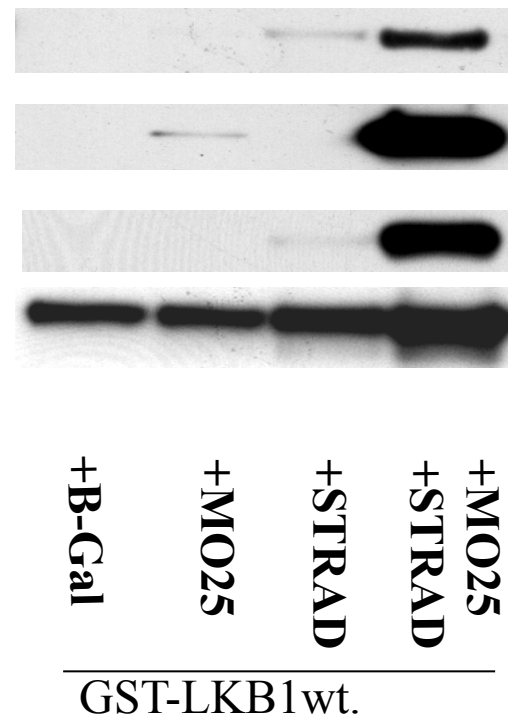

Supplement: Supplementary file 1 [file nutrients-09-00751-s001.pdf]
